# *CHRNA3* Polymorphism Modifies Lung Adenocarcinoma Risk in the Chinese Han Population

**DOI:** 10.3390/ijms15045446

**Published:** 2014-03-28

**Authors:** Ping He, Xue-Xi Yang, Xuan-Qiu He, Jun Chen, Fen-Xia Li, Xia Gu, Ju-Hong Jiang, Hui-Ying Liang, Guang-Yu Yao, Jian-Xing He

**Affiliations:** 1 Department of Pathology, the First Affiliated Hospital of Guangzhou Medical University, Guangzhou 510120, China; E-Mails: hp5567@163.com (P.H.); guxia1373@sohu.com (X.G.); juhongjiang@163.com (J.-H.J.); 2 School of Biotechnology, Southern Medical University, Guangzhou 510515, China; E-Mail: yxxzb@sohu.com; 3 The First Clinical College, Southern Medical University, Guangzhou 510515, China; E-Mail: lmsh815@163.com; 4 Breast Center, Nanfang Hospital, Southern Medical University, Guangzhou 510515, China; E-Mails: chenjun@163.com (J.C.); ygy531@163.com (G.-Y.Y.); 5 Da An Gene Co., Ltd. of Sun Yat-sen University, Guangzhou 510665, China; E-Mail: lifenxia@gmail.com; 6 Department of Primary Public Health, Guangzhou Center for Disease Control and Prevention, Guangzhou 510440, China; E-Mail: lianghuiying@163.com; 7 Department of Cardiothoracic Surgery, the First Affiliated Hospital of Guangzhou Medical University, Guangzhou 510120, China; 8 State Key Laboratory of Respiratory Diseases, Guangzhou Institute of Respiratory Disease, the First Affiliated Hospital of Guangzhou Medical University, Guangzhou 510120, China

**Keywords:** lung adenocarcinoma, single nucleotide polymorphism, *CHRNA3*, female nonsmokers

## Abstract

Recent genome-wide association studies (GWASs) have identified 15q25.1 as a lung cancer susceptibility locus. Here, we sought to explore the direct carcinogenic effects of genetic variants in this region on the risk of developing lung adenocarcinoma (ADC). Five common SNPs (rs8034191, rs16969968, rs1051730, rs938682, and rs8042374) spanning the 15q25.1 locus were assayed in a case-control study examining a cohort of 301 lung ADCs and 318 healthy controls. Stratification analysis by gender, smoking status, and tumor, node, metastasis (TNM) classification, was performed. In addition, sections from ADC tissue and normal tissue adjacent to tumors were stained with an anti-CHRNA3 (cholinergic receptor nicotinic α3) antibody by immunohistochemistry in 81 cases. Our results demonstrate that rs8042374, a variant of the *CHRNA3* gene, is associated with an increased risk of ADC with an OR of 1.76 (95% CI: 1.17–2.65, *p* = 0.024). This variant was linked to a greater risk of ADC in female nonsmokers (OR (95% CI): 1.81 (1.05–3.12), *p* = 0.032) and female stage I + II cases (OR (95% CI): 1.92 (1.03–3.57), *p* = 0.039). Although located within the same gene, rs938682 showed protective effects for smokers, stage III + IV cases, and male stage III + IV cases. Additionally, the CHRNA3 protein level in ADC tissue was slightly higher than in the surrounding normal lung tissue, based on immunohistochemical analysis. Our results suggest that the *CHRNA3* polymorphism functions as a genetic modifier of the risk of developing lung ADC in the Chinese population, particularly in nonsmoking females.

## 1. Introduction

Primary lung cancer is the most common cancer, responsible for over one million deaths worldwide annually [1]. In China, its morbidity and mortality have increased rapidly in the last three decades, which is attributed to complex interactions between environmental and genetic risk factors [2]. Although over 80% of lung cancer deaths are tobacco-related, only a small fraction of smokers (usually <20%) develop lung cancer [2], indicating that inter-individual differences in the rate of genetic variations may be related to lung carcinogenesis. Single-nucleotide polymorphisms (SNPs) are the most common and stable markers of human genetic variation, and may be associated with the risk of a variety of cancers, including that of the lung [3,4]. Recently, much evidence of a direct/indirect effect of polymorphic variation at 15q25.1 on lung cancer risk has been provided by genome-wide association studies (GWASs) and segregation analyses in European, American and Asian populations [5–8]. For example, three SNPs (rs8034191, rs1051730 and rs16969968) at 15q25.1 were consistently strongly associated with lung cancer risk in both smokers and nonsmokers in populations of European descent [5,8]. However, the results failed to be replicated in Korean, Japanese and Chinese populations, partly because the minor allelic frequencies (MAF) were extremely low in Asian populations according to the HapMap database [9–11]. It appears that genetic determinants for the risk of lung cancer are heterogeneous at both the allelic and locus levels among populations of various ethnicities.

The chromosome region 15q25.1 contains the *AGPHD1* (aminoglycoside phosphotransferase domain containing 1), *CHRNA5* and *CHRNA3* (cholinergic receptor nicotinic α5 and 3) gene cluster, which express nicotinic acetylcholine receptor subunits (nAChRs) [12,13]. Activation of nAChRs facilitates the outgrowth of cells with genetic damage and promotes cell proliferation, migration, invasion, and angiogenesis, which stimulates the development of lung cancer cells and suppresses apoptosis by acting as tumor promoters [14]. However, debate remains on whether the association is established through a direct effect on a gene that causes lung cancer or facilitated by means of an indirect effect leading to nicotine addiction. Additionally, the very high linkage disequilibrium (LD) in the 15q25.1 locus, as documented in the literature [5–8], has raised the question as to whether all SNPs identified in this locus are causative variants for lung cancer. Therefore, studies aiming to define the biological effects of these SNPs may provide a mechanistic understanding of genetic susceptibility to lung cancers.

Although the histological spectrum of lung cancer demonstrates geographic variations, there has been a major global trend towards a decrease in squamous cell carcinoma (SCC) and a marked increase in adenocarcinoma (ADC) [15]. Moreover, the majority of ADCs occur in female nonsmokers [16], suggesting that their mechanisms of carcinogenesis differ from the more common tobacco-dependent forms of lung cancer. Therefore, we sought to identify the genetic variants that modify ADC risk after dividing subjects according to gender and smoking status. A case-control study was performed to examine five common SNPs (rs8034191, rs16969968, rs1051730, rs938682, and rs8042374) on 15q25.1 in a population of Chinese ancestry.

## 2. Results

The case-control study consisted of 301 ADC cases and 318 cancer-free controls in a Chinese Han population (Table 1). The mean ages of all control patients were 56.1 ± 12.0 years (range 19–75 years) and 59.6 ± 10.8 years (range 23–84 years) at diagnosis/selection. Subjects comprised 156 (49%) males and 162 (51%) females in the control group, and 147 (49%) males and 154 (51%) females in the case group. Seventy-three percent of subjects did not smoke, compared with 27% that did. One hundred and eighty-three (61%) ADC patients presented at stage I + II, and 118 (39%) presented at stage III + IV according to the TNM classification.

All subjects were genotyped for six SNPs (rs8034191T/C, rs16969968G/A, rs1051730C/T, rs938682T/C, rs12914385T/C, and rs8042374G/A) in 15q25.1. The SNPs were in Hardy-Weinberg equilibrium (HWE) both in controls and cases (*p* > 0.05), except for rs12914385, which was excluded from subsequent analyses. Of the five successfully genotyped SNPs, a highly significant association with ADC risk was found for heterozygotes (GA) of rs8042374G/A in the *CHRNA3* gene, with an odds ratio (OR) = 1.76 (95% confidence interval (CI), 1.17–2.65; *p* = 0.024) in the codominant model, as well as a more highly significant association in the overdominant model as the fitting model with an OR = 1.71 (95% CI, 1.15–2.54; *p* = 0.008) compared with the genotypes (GG/AA) (Table 2). Another SNP in *CHRNA3*, rs938682T/C, showed a marginally significant association for variant genotypes (TC/CC) compared with the wild-type genotype (TT), with an OR = 0.68 (95% CI, 0.45–1.02; *p* = 0.063) in the dominant model as the fitting model. The other three SNPs showed no polymorphism and no significant association with ADC risk.

**Figure 1.**
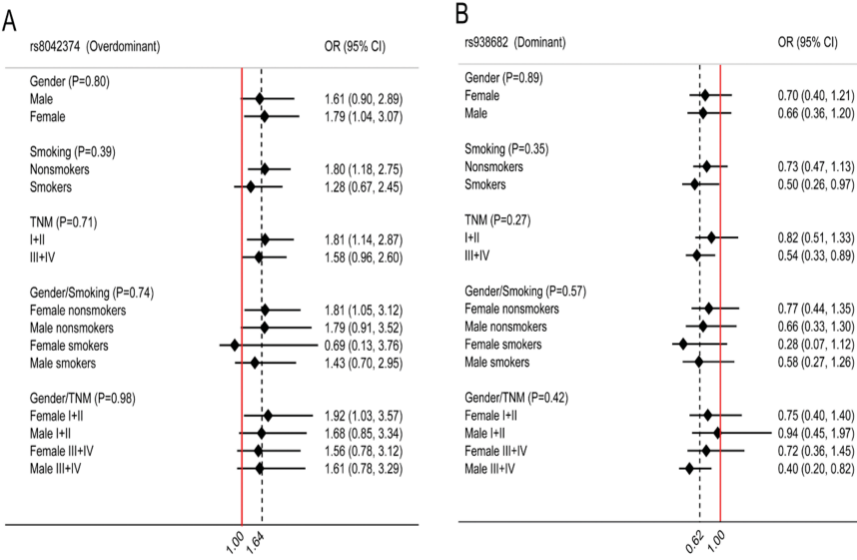
Association of lung ADC risk with two SNPs stratified by gender, smoking status, and TNM stage. (**A**) rs8042374; (**B**) rs938682.

Furthermore, we conducted gene-environment interaction analyses for the two significant loci in the respectively fitting models (Supplementary Information, Table S1). The GA-rs8042374 exhibited strong significant associations with the stratified factors, including female gender, nonsmokers, stage I + II cases, female nonsmokers, and female stage I + II cases in the overdominant model, with ORs = 1.79 (95% CI, 1.04–3.07), 1.80 (95% CI, 1.18–2.75), 1.81 (95% CI, 1.14–2.87), 1.81 (95% CI, 1.05–3.12), and 1.92 (95% CI, 1.03–3.57), respectively (Figure 1A). Interestingly, the variant genotypes (TC/CC) of rs938682 showed protective effects for smokers, stage III + IV cases and male stage III + IV cases in the dominant model, with ORs = 0.50 (95% CI, 0.26–0.97), 0.54 (95% CI, 0.33–0.89) and 0.40 (95% CI, 0.20–0.82), respectively (Figure 1B).

Haplotype analysis revealed that subjects carrying the TGCTA haplotype had a 1.43-fold (*p* = 0.037, Table 3) increased risk of ADC compared to the reference haplotype (TGCCG).

**Figure 2.**
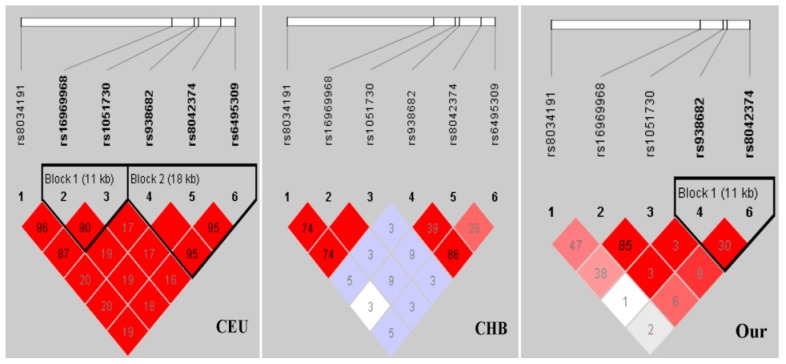
Linkage disequilibrium (LD) patterns of five SNPs in 15q25.1 among various populations. Numbers inside the boxes represent *r*^2^ values for LD. Colors indicate the strength of LD between pair-wise combinations of SNPs (white, low LD; red, high LD).

We next examined the LD among these five SNPs located in 15q25.1 in the HapMap CEU and CHB population and our data. The LD structure (rs938682/rs8042374) showed high correlation in CEU (*r*^2^ = 1.00), but low correlation in CHB and our data (*r*^2^ = 0.39 and 0.30, respectively; Figure 2).

**Figure 3.**
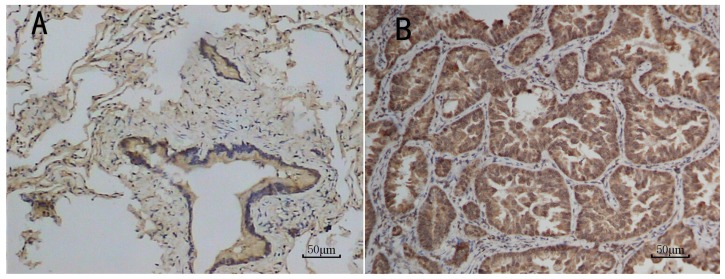
Immunohistochemical analysis of CHRNA3 protein in normal lung and lung ADC tissue, showing a cytoplasmic staining pattern. In normal lung tissue, immunoreactivity was detected in alveolar and bronchial cells (**A**, ×200); In ADC, immunoreactivity was detected in tumor cells, but not in stromal cells (**B**, ×200).

**Figure 4.**
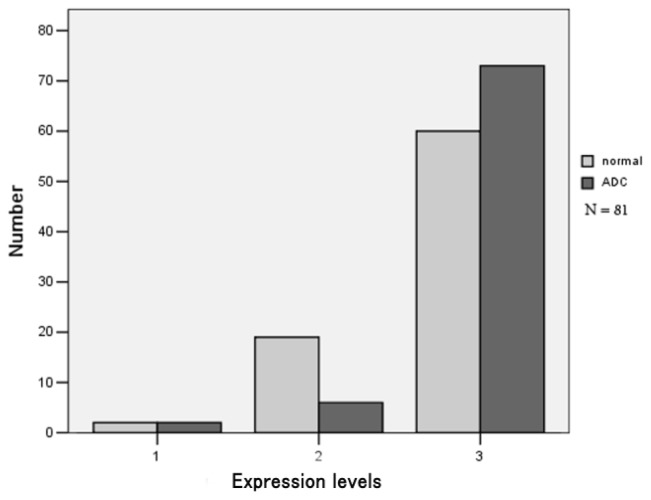
CHRNA3 protein levels in ADC and normal lung.

Eighty-one cases of ADC specimens were collected for immunohistochemical analysis. The results readily revealed the expression of CHRNA3 protein mainly in the cytoplasm in lung ADC and surrounding normal lung tissue. Immunoreactivity in normal tissue was apparent in alveolar cells, as well as bronchiolar cells (Figure 3A). In lung ADC, CHRNA3 protein was detected in tumor cells but not in stromal cells (Figure 3B). Statistical analysis showed a significant difference in expression levels, with higher immunostaining intensity in ADC tissue than in normal lung tissue (*p* = 0.001; Figure 4). However, further analysis of *CHRNA3* variant genotypes and protein expression did not indicate a significant correlation (data not shown).

## 3. Discussion

The five SNPs examined in this study are located at chromosome 15q25.1, which contains the common *AGPHD1*, *CHRNA5* and *CHRNA3* genes. Our results demonstrate that rs8034191, rs16969968 and rs1051730, variants in *AGPHD1*, *CHRNA5* and *CHRNA3*, respectively, display non-significant associations with ADC. However, rs8042374, a variant of the *CHRNA3* gene, is associated with an increased risk of ADC, especially in subtypes stratified by female nonsmokers and female stage I + II cases. In contrast, another SNP within *CHRNA3*, rs938682, appears to be associated with a reduced risk of ADC.

*CHRNA3* encodes nAChRs, which is present either in neurons or in lung epithelial cells and lung cancer cell lines. nAchRs bind not only to nicotine but also nicotine-derived nitrosamines, which are potent lung carcinogens [17,18]. These carcinogens might activate signal transduction through nAChRs expressed in human airway epithelial cells, which could cause loss of contact inhibition and resistance to apoptosis in cells, and also facilitate neoplastic transformation [14]. The significant SNP in the present study, rs8042374, localizes to intron 4 of *CHRNA3*; this was first confirmed with increased lung cancer risk in a British GWAS [19], but was not replicated in another study of ADC among British residents of European ancestry [20]. This suggests that genetic and/or environmental factors may account for the observed differences. Moreover, there is controversy as to whether SNPs in the *CHRNA3* gene have a direct carcinogenic effect on lung cancer risk or impact indirectly through smoking. Although many studies have demonstrated that lung cancer risk associated with SNPs is mediated through the propensity to smoke, and hence a higher exposure to smoking-related carcinogens [21], it does not exclude the possibility that the variants also have a direct effect on lung cancer risk in nonsmokers [8].

There is strong evidence to support the notion that ADC arising in nonsmokers is distinct from the more common tobacco-dependent forms of lung cancer in terms of genetic variations. For example, three major genes involved in the pathogenesis of lung cancers, *EGFR*, *KRAS* and *TP53*, exhibit striking differences in terms of the molecular alterations found in ADC and tobacco-dependent forms of lung cancer, providing evidence that these cancers arise through different molecular mechanisms [16].

Our results demonstrate that the rs8042374 variant genotype (GA) is associated with an increased risk of ADC (OR: 1.76, 95% CI: 1.17–2.65). Specifically, statistically significant gene–environment interactions between the GA variant genotype and female, nonsmokers, stage I + II cases, female nonsmokers and female I + II cases were observed, implying that carriers of the rs8042374 variant genotype (GA) have a greater risk of developing ADC than those who carry the wild-type allele, irrespective of tobacco consumption. The SNP rs938682 displayed no significant association with ADC or SCC in Chinese nonsmokers according to other researchers [22]. However, the rs938682 TC/CC variant genotypes were marginally significantly associated with the protective effect of ADC, with an OR = 0.68 (95% CI, 0.45–1.02) in our study. Subtype analysis confirmed a contribution to this protective effect to smokers and Stage III + IV cases and male III + IV cases, which indicates that carriers with a copy of the rs938682 variant genotype have lower ADC-associated morbidity than those with the wild-type genotype.

Interestingly, a GWAS based on the Chinese population confirmed another functional variant—rs6495309C > T in the *CHRNA3* promoter, which resulted in increased CHRNA3 expression through transcriptional factor Oct-1 binding ability [11]. It is unfortunate that we did not genotype the rs6495309 for mis-selection. However, according to the Haploview analysis, the LD pattern showed a high correlation between rs6495309–rs938682 in the HapMap CEU and CHB populations (*r*^2^ = 0.95, 0.86, respectively), as well as a high correlation between rs6495309–rs8042374 in the CEU but a low correlation in the CHB (*r*^2^ = 0.95, 0.26, respectively; Figure 2), suggesting that rs938682 and/or rs8042374 may tag rs6495309, and vice versa. Further fine-mapping studies are needed for the interpretation and genetic characterization of these associations.

To evaluate the differential expression of CHRNA3 between ADC and normal lung subjects, immunohistochemical localization of the *CHRNA3*-encoded protein was performed in paired ADC tissue and normal tissue adjacent to tumors. Immunohistochemical results revealed moderate or strong expression of CHRNA3 protein in both tissues; expression was significantly higher in ADC than in normal lung tissue (*p* = 0.001), although the correlation analysis did not reveal an association between variant genotypes and CHRNA3 protein expression. In contrast, Falvella *et al.* reported downregulated *CHRNA3* mRNA levels in ADC [23] and Lam *et al.* detected no differences in *CHRNA3* expression in non-small-cell lung cancer cell lines or in tumor compared with normal tissue [24]. Identification of causal genetic variants is challenging and requires further extensive genetic and biological investigations.

There are some limitations in our study. First, the number of subjects enrolled in this study is relatively small. Although the analysis showed insignificant association for some of the tested SNPs, those are rare genetic variants in CHB population and we could not rule out that the weak association is the result of type II error. In addition, precise data regarding smoking dose and duration were not available and direct associations of risk alleles with tobacco consumption were not confirmed. Finally, the potential functional SNP rs6495309C > T should have been included in our analysis.

A major strength of our study was that the case group was composed entirely of ADC patients, allowing for a better understanding of the mechanisms underlying this type of lung cancer. Secondly, the gene-environmental interaction analyses stratified by gender, smoking status, and TNM classification enabled screening for potential environmental risk factors.

## 4. Experimental Section

### 4.1. Population

All eligible subjects were members of the Chinese Han population, and were recruited from the outpatient and inpatient clinics of the First Affiliated Hospital, Guangzhou Medical University, Guangzhou, Guangdong Province, China, between September 2011 and August 2012. The 301 patients enrolled in the study were those with histologically confirmed ADC without a history of other cancers. Routine tumor, node, metastasis (TNM) classification was performed according to the new staging protocol initiated by the International Association for the Study of Lung Cancer in 2009 [25]. The control population was comprised of 318 individuals selected randomly from the hospital’s Health Examination Center during the same time period, and was frequency matched to patients by gender and age. The control group comprised individuals with no history of cancer and without lung-related diseases to avoid probable interference from overlapping genes. All subjects provided standard informed consent, approved by the Guangzhou Medical University Ethics Committee. Smoking history was obtained through an interview using a questionnaire. Individuals who had smoked fewer than 100 cigarettes in their lifetime were defined as nonsmokers; all other individuals were defined as smokers, including those who had been smoke-free for >1 year.

### 4.2. SNP Selection

Six SNPs in 15q25.1 (rs8034191 in *AGPHD1*; rs16969968 in *CHRNA5*; rs1051730, rs938682, rs12914385, and rs8042374 in *CHRNA3*) were selected based upon GWAS and meta-analyses of multiple populations [5–8,18–21]. However, since the genotypic distribution deviated from Hardy-Weinberg equilibrium in the patient samples and in the control group, rs12914385 was excluded from the following analyses.

### 4.3. DNA Extraction and Genotyping

Genomic DNA was extracted from peripheral blood using a commercial blood DNA kit (TIANamp Genomic DNA Purification Kit; Tiangen Biotech, Beijing, China), according to the manufacturer’s instructions, and stored at −70 °C until use.

The selected SNPs were genotyped using the SEQUENOM MassARRAY matrix-assisted laser desorption ionization-time of flight mass spectrometry platform (Sequenom, San Diego, CA, USA). Primers for polymerase chain reaction (PCR) and single-base extension were designed using Assay Designers software version 3.1 (Sequenom, San Diego, CA, USA). SNPs were genotyped using Sequenom MassARRAY (Sequenom, San Diego, CA, USA) genotyping technology and amplified by multiplex PCR using a standard PCR protocol, in accordance with the manufacturer’s instructions. Genomic amplification products were then purified with shrimp alkaline phosphatase to neutralize any unincorporated deoxynucleotide triphosphates (dNTPs), followed by a single-base extension reaction using the iPLEX enzyme and mass-modified terminators. Products of the iPLEX reaction were desalted and transferred onto a SpectroCHIP by the MassARRAY nanodispenser, which was then assessed by the MassARRAY analyzer by combining base calling with the clustering algorithm.

### 4.4. Immunohistochemistry

Paraffin-embedded tissue sections of ADC and surrounding normal lung tissue from patients were retrieved from the archives of the Department of Pathology, First Affiliated Hospital, Guangzhou Medical University. Histological sections were immunostained with an anti-CHRNA3 antibody (ab103956, diluted 1:50; Abcam, Inc., Cambridge, MA, USA) using standard methods after antigen retrieval in ethylene diamine tetraacetic acid (EDTA, pH 8.0) at 100 °C for 20 min. Immunoreactive signals were detected with Chem-Mate DAB (Dako, K5007; Glostrup, Denmark). All immunostaining results were evaluated by two pathologists independently. Immunoreactivity scores were determined based upon both staining intensity and density. Staining intensity was scored as follows: 0 (negative), 1 (weakly positive), 2 (moderately positive), and 3 (strongly positive). The percentage of CHRNA3-positive cells was also scored according to the following four levels: 1 was given for 0 to 10%, 2 for 11% to 50%, 3 for 51% to 75%, and 4 for 76% to 100%. The combined scores were recorded and graded as follows: −, 0; +, 1–2; ++, 3–5; +++, 6–7.

### 4.5. Statistical Analysis

Hardy-Weinberg equilibrium (HWE) testing was carried out for all selected SNPs. Pearson’s χ^2^ test was used to examine the differences in the distributions of genotypes between cases and controls. An unconditional logistic regression was used to calculate ORs and 95% CIs, which were used to estimate the association between a single locus and ADC risk, with adjustment for age and gender to control for confounding effects. ORs for each genotype were calculated in codominant (*i.e*., AA *versus* Aa *versus* aa; A: major allele, a: minor allele), overdominant (*i.e*., AA/aa *versus* Aa) or dominant (*i.e*., AA *versus* Aa/aa) models, which acted as a more fitting model. To minimize the confounding effects of gender, smoking status and pathological staging, we performed the interaction analyses for each SNP on the confounding factors individually (comparing subtype cases to all controls). These statistical analyses were performed using the SPSS 13.0 statistical software (SPSS Inc., Chicago, IL, USA). Haplotypes were estimated using the web-based tool SNPstats (http://bioinfo.iconcologia.net/SNPStats). The Haploview software version 4.1 was used to calculate *r*^2^ values using the expectation-maximization algorithm. Immunohistochemical staining results were scored, and the significance of differences evaluated using Wilcoxon’s signed-ranks test. Correlations between SNP genotypes and gene expression were assessed with Spearman’s rank-order correlation coefficient. A *p* value <0.05 for an OR was used as the criterion of statistical significance, and all statistical tests were two-sided.

## 5. Conclusions

In conclusion, our data suggest that the *CHRNA3* gene contributes significantly to ADC in the Chinese Han population, especially the risk effect of rs8042374 for female nonsmokers and female I + II cases, as well as the protective effect of rs938682 for smokers, stage III + IV cases and male III + IV cases. Additionally, the CHRNA3 protein level in ADC tissue was slightly higher than that in normal tissue adjacent to tumors, based on immunohistochemical analysis. However, the genetic variants accounted for only a small proportion of the total genetic susceptibility to ADC. Studies of larger population-based case series of ADC or other histopathological lung cancers are needed to identify the underlying biological mechanism.

## Supplementary Information



## Figures and Tables

**Table 1.** Characteristics of controls and cases in a Chinese Han population.

Variable	Control	Case	*p* Value^b^
Total	318	301	
Age (mean ± SD, years)	56.1 ± 12.0 (19–75)	59.6 ± 10.8 (23–84)	0.002

Gender			0.956

Male, n (%)	156 (49%)	147 (49%)	
Female, n (%)	162 (51%)	154 (51%)	

Smoking status			0.974

Nonsmokers	231 (73%)	219 (73%)	
Smokers	87 (27%)	82 (27%)	

TNM^a^			

I + II		183 (61%)	
III + IV		118 (39%)	

a Tumor, node, metastasis;

b *t*-Tests and chi-square tests were used to test for difference between cases and controls.

**Table 2.** Associations between candidate SNPs in 15q25.1 and risk of lung ADC in a Chinese population.

					Codominant Model		Fitting Model	
						
Gene	SNP	Position	Alleles^a^	MAF^b^	Heterozygote OR (95% CI)^c^	Homozygote OR (95% CI)^c^	OR (95% CI)^c^	*p* Value
*AGPHD1*	rs8034191	78806023	T/C	0.03/0.04	1.31 (0.63–2.73)	No polymorphism		
*CHRNA5*	rs16969968	78882925	G/A	0.04/0.04	1.14 (0.57–2.27)	No polymorphism		
*CHRNA3*	rs1051730	78894339	C/T	0.04/0.04	1.17 (0.58–2.35)	No polymorphism		
	rs938682	78896547	T/C	0.46/0.43	0.67 (0.43–1.03)	0.71 (0.42–1.21)	0.68 (0.45–1.02)	0.063^d^
	rs8042374	78908032	G/A	0.27/0.28	1.76 (1.17–2.65)	1.28 (0.59–2.75)	1.71 (1.15–2.54)	**0.008**^e^

a Reference allele/risk allele;

b Control/case;

c Adjusted for age and gender;

d Dominant model for rs938682;

e Overdominant model for rs8042374. Bold mean *p* < 0.05.

**Table 3.** Haplotype analyses of five SNPs in 15q25.1 with lung adenocarcinoma risk.

Haplotype	rs8034191	rs16969968	rs1051730	rs938682	rs8042374	Frequency^a^	OR (95% CI)^b^	*p* Value
1	T	G	C	C	G	0.46/0.43	1.00 (reference)	
2	T	G	C	T	G	0.27/0.27	1.02 (0.73–1.42)	0.910
3	T	G	C	T	A	0.23/0.25	1.43 (1.02–2.00)	**0.037**

a Controls/Cases;

b odds ratio (OR) adjusted for age. Bold mean *p* < 0.05.
