# Supplementary material for: CHRNA3 Polymorphism Modifies Lung Adenocarcinoma Risk in the Chinese Han Population
Source: Int J Mol Sci. 2014 Mar 28;15(4):5446–57. doi: 10.3390/ijms15045446 (PMC4013574; doi:10.3390/ijms15045446)
Supplement: Supplementary file 1 [file ijms-15-05446-s001.pdf]

Supplementary Information

Table S1. Association of the risk of lung adenocarcinoma with two SNPs stratified by gender, smoking status and TNM staging.

Fitting Model	rs8042374		rs938682	
	Overdominant		Dominant	
	OR (95% CI) [a]	*p*	OR (95% CI) [a]	*p*
Female (*n* = 154)	1.79 (1.04–3.07)	**0.033**	0.70 (0.40–1.21)	0.200
Male (*n* = 147)	1.61 (0.90–2.89)	0.110	0.66 (0.36–1.20)	0.170
Nonsmokers (*n* = 219)	1.80 (1.18–2.75)	**0.006**	0.73 (0.47–1.13)	0.160
Smokers (*n* = 82)	1.28 (0.67–2.45)	0.460	0.50 (0.26–0.97)	**0.042**
I + II (*n* = 183)	1.81 (1.14–2.87)	**0.011**	0.82 (0.51–1.33)	0.420
III + IV (*n* = 118)	1.58 (0.96–2.60)	0.072	0.54 (0.33–0.89)	**0.016**
Female nonsmokers (*n* = 144)	1.81 (1.05–3.12)	**0.032**	0.77 (0.44–1.35)	0.360
Male nonsmokers (*n* = 75)	1.79 (0.91–3.52)	0.091	0.66 (0.33–1.30)	0.230
Female smokers (*n* = 10)	0.69 (0.13–3.76)	0.670	0.28 (0.07–1.12)	0.068
Male smokers (*n* = 72)	1.43 (0.70–2.95)	0.330	0.58 (0.27–1.26)	0.170
Female I + II (*n* = 96)	1.92 (1.03–3.57)	**0.039**	0.75 (0.40–1.40)	0.360
Male I + II (*n* = 87)	1.68 (0.85–3.34)	0.130	0.94 (0.45–1.97)	0.870
Female III + IV (*n* = 58)	1.56 (0.78–3.12)	0.210	0.72 (0.36–1.45)	0.360
Male III + IV (*n* = 60)	1.61 (0.78–3.29)	0.200	0.40 (0.20–0.82)	**0.012**

[a] Adjusted by age and gender. Bold mean $p < 0.05$.

© 2014 by the authors; licensee MDPI, Basel, Switzerland. This article is an open access article distributed under the terms and conditions of the Creative Commons Attribution license (http://creativecommons.org/licenses/by/3.0/).
